# Enhanced Cerebral Hemodynamics and Cognitive Function Via Knockout of Dual-Specificity Protein Phosphatase 5

**DOI:** 10.26502/fjppr.070

**Published:** 2023-05-12

**Authors:** Huawei Zhang, Jane J. Border, Xing Fang, Yedan Liu, Chengyun Tang, Wenjun Gao, Shaoxun Wang, Seung Min Shin, Ya Guo, Chao Zhang, Ezekiel Gonzalez-Fernandez, Hongwei Yu, Peng Sun, Richard J. Roman, Fan Fan

**Affiliations:** 1 Pharmacology &Toxicology, University of Mississippi Medical Center, Jackson, MS, USA; 2 Neurosurgery, Affiliated Hospital of Qingdao University, Qingdao, China; 3 Physiology, Medical College of Georgia, Augusta University, Augusta, GA, USA; 4 Anesthesiology, Medical College of Wisconsin, Milwaukee, WI, USA

**Keywords:** *Dusp5*, cerebral hemodynamics, PKC, ERK, AD/ADRD

## Abstract

Alzheimer’s Disease (AD) and Alzheimer’s Disease-Related Dementias (ADRD) are neurodegenerative disorders. Recent studies suggest that cerebral hypoperfusion is an early symptom of AD/ADRD. Dual-specificity protein phosphatase 5 (DUSP5) has been implicated in several pathological conditions, including pulmonary hypertension and cancer, but its role in AD/ADRD remains unclear. The present study builds on our previous findings, demonstrating that inhibition of ERK and PKC leads to a dose-dependent dilation of the middle cerebral artery and penetrating arteriole, with a more pronounced effect in *Dusp5* KO rats. Both ERK and PKC inhibitors resulted in a significant reduction of myogenic tone in vessels from *Dusp5* KO rats. *Dusp5* KO rats exhibited stronger autoregulation of the surface but not deep cortical cerebral blood flow. Inhibition of ERK and PKC significantly enhanced the contractile capacity of vascular smooth muscle cells from both strains. Finally, a significant improvement in learning and memory was observed in *Dusp5* KO rats 24 hours after initial training. Our results suggest that altered vascular reactivity in *Dusp5* KO rats may involve distinct mechanisms for different vascular beds, and DUSP5 deletion could be a potential therapeutic target for AD/ADRD. Further investigations are necessary to determine the effects of DUSP5 inhibition on capillary stalling, blood-brain barrier permeability, and neurodegeneration in aging and disease models.

## 1. Introduction

Recent studies have provided evidence suggesting that brain hypoperfusion, a condition characterized by a reduction in cerebral blood flow (CBF), can contribute to neurodegeneration, leading to cognitive impairments in individuals with Alzheimer’s Disease (AD) and Alzheimer’s Disease-Related Dementias (ADRD) [1, 2]. Human studies have identified a strong correlation between the severity of CBF reduction and the extent of cognitive deficits, highlighting the importance of addressing CBF reduction as one of the early symptoms of AD/ADRD [3, 4]. Despite the significant attention on the impact of CBF reduction on cognitive function and brain pathology in AD/ADRD, the genetic, molecular, and cellular mechanisms underlying this relationship remain poorly understood.

Several factors may contribute to reduced CBF in individuals with AD/ADRD. Studies have identified impaired myogenic response and autoregulation, neurovascular dysfunction, capillary stalling, blood-brain barrier (BBB) leakage, and diminished venous and neurovascular-glymphatic function as potential vascular mechanisms [5–9]. Mural cells on the vessel wall, such as vascular smooth muscle cells (VSMCs) and pericytes, play a crucial role in regulating vascular diameter and maintaining constant brain perfusion. Studies have indicated that impairment to the VSMCs and pericytes that are positive for alpha-smooth muscle actin can lead to a decline in the myogenic response of cerebral arteries and arterioles, including the middle cerebral arteries (MCAs) and penetrating arterioles (PAs). This, in turn, can negatively affect the ability of CBF autoregulation in individuals with AD/ADRD [7, 10–12].

Dual-specificity protein phosphatase 5 (DUSP5) is a versatile phosphatase that can dephosphorylate both threonine and tyrosine residues, making it a crucial regulator of signaling pathways. DUSP5 functions as a negative regulator of the mitogen-activated protein kinase (MAPK) superfamily by targeting extracellular signal-related kinase (ERK), a key signaling molecule involved in diverse cellular processes such as proliferation, differentiation, and survival [13, 14]. DUSP5 interacts with ERK between feedback loops, resulting in the inactivation and nuclear anchoring of ERK1/2. Paradoxically, this process enhances cytoplasmic ERK activity, while cytoplasmic activation of ERK facilitates nuclear translocation [15–18]. DUSP5 has been shown to directly target the tumor suppressor protein p53, and its dysregulation has been implicated in tumorigenesis and cancer progression in numerous human malignancies [19–21]. In addition, DUSP5 also plays a role in the induction of cell death in response to endoplasmic reticulum (ER) stress [22]. Interestingly, DUSP5 expression has been shown to be upregulated by angiotensin II (ANG II) in primary rat aortic VSMCs, [23] but DUSP5 serves as a negative regulator of ANG-II-mediated cell proliferation in VSMCs from de-identified patients with pulmonary arterial hypertension [24]. The Harder group has reported [25] that knockdown of *Dusp5* in isolated and cultured cerebral arteries results in an enhanced myogenic response and increased phosphorylation of protein kinase C (PKC)-βII. We have generated *Dusp5* knockout (KO) rats and confirmed that myogenic response and blood flow autoregulation were enhanced in both cerebral and renal circulations. This enhancement was associated with increased phosphorylation of PKCα and ERK1/2 [26–28]. In the kidney, *Dusp5* KO has been shown to protect against hypertension-induced impaired renal hemodynamics, attenuate elevated glomerular capillary permeability, renal fibrosis, medullary protein cast formation, macrophage infiltration, and epithelial-mesenchymal transformation [27, 29]. More recently, we have discovered that *Dusp5* KO rats exhibited eutrophic vascular hypotrophy, higher myogenic tones, greater compliance, improved distensibility, and reduced stiffness in PAs and renal interlobular arterioles [28]. Furthermore, VSMCs isolated from *Dusp5* KO rats displayed a stronger contractile capability.

DUSPs are also known to play crucial roles in the pathophysiology of neurodegenerative diseases, such as AD/ADRD and Parkinson’s disease [30]. Previous reports have suggested that *DUSP5* is one of the transcripts that regulate hippocampal dentate gyrus plasticity [31]. Moreover, *DUSP5* is located within a locus that shows strong evidence of association with late-onset AD [32]. A weighted gene co-expression network analysis of the microarray dataset downloaded from Gene Expression Omnibus (GEO), [33] which included 188 control human cerebral cortex samples and 176 patients with late-onset AD, revealed that *DUSP5* is one of the 16 hub genes in the AD *APOE* ε4 non-carriers module. This finding suggests that DUSP5 may be involved in AD pathogenesis through mechanisms independent of β-amyloid (Aβ) aggregation, neurofibrillary tangle formation, and synaptic plasticity impairment, which are observed in AD patients carrying *APOE* ε4 [34]. In this study, our objective was to expand our previous findings by examining the effects of ERK and PKC inhibitors on pressure-induced myogenic responses of MCA and PA isolated from WT and *Dusp5* KO rats. We also evaluated the effects of *Dusp5* KO on autoregulation of the surface and deep cortical in response to perfusion pressure ranging from 40 to 180 mmHg. Additionally, we investigated the effects of ERK and PKC inhibition on the contractile capability of cerebral VSMCs isolated from wildtype (WT) and *Dusp5* KO rats and the effects of *Dusp5* KO on the cerebrovascular responsiveness to a thromboxane A2 (TXA_2_) agonist. Finally, we compared cognitive function between WT and *Dusp5* KO rats.

## 2. Material and Methods

### 2.1 General

In this project, the FHH.1^BN^ congenic rat strain (FHH.1^BN-(*D1Rat09-D1Rat225*)/Mcwi^), which had previously been generated, was defined as WT rats [35–38]. The experiments were conducted in WT and *Dusp5* KO rats that were on the FHH.1^BN^ background (FHH-Chr 1^BN^-*Dusp5*^em1Mcwi^) [26–28]. WT and *Dusp5* KO rats aged 36 weeks were used for behavior evaluation, 8–12 weeks for vascular studies, and 3-week-old rats for primary cerebral VSMC isolation. The optimal time and dose points of inhibitors of PKC and ERK for their effects on myogenic reactivity in freshly isolated MCAs were evaluated in 35 Sprague-Dawley (SD) rats, aged between 8–12 weeks. These rats were bred and maintained under a controlled photoperiod (12-hour light–dark cycle) with unlimited *ad libitum* access to water and a standard diet (Teklad rodent diet 8604, Envigo, Indianapolis, IN) at the University of Mississippi Medical Center. All protocols in this study were approved by the Institutional Animal Care and Use Committee at the UMMC, following the guidelines of the American Association for the Accreditation of Laboratory Animal Care.

A selective ERK1/2 inhibitor FR180204 (FR; sc-203945, Santa Cruz, Dallas, Texas), a selective PKC inhibitor bisindolylmaleimide III (BIM; sc-311291, Santa Cruz), and a thromboxane A_2_ agonist U46619 (ab144540, Abcam, Cambridge, MA) were dissolved in Dimethyl sulfoxide (DMSO) for stock solutions at 1 mM, 300 μM, and 1 mM, respectively. The stock solutions were aliquoted in tightly sealed Eppendorf tubes at −20 °C for one month. The working solutions were freshly diluted to 1,000 X of desired concentrations on the same day of experiments. The same value of DMSO (0.1%) was used as the vehicle.

### 2.2 Vessel Preparation

The MCA and PA were isolated following the protocol we described previously [10–12, 28, 36, 39]. Briefly, the rats were euthanized with 4% isoflurane. The brains were quickly collected and placed in a dish containing ice-cold calcium-free physiological salt solution (PSS_0Ca_), made with 119 NaCl, 4.7 KCl, 1.17 MgSO_4_, 0.03 EDTA, 18 NaHCO_3_, 5 HEPES, 1.18 NaH_2_PO_4_, 10 glucose (in mM, pH 7.4) [40–42]. Branch-free M2 segments of MCA and the PA downstream of the lenticulostriate arteries [43–46] were dissected in an ice-cold PSS_0Ca_ supplemented with 1% bovine serum albumin and placed in ice-cold PSS_0Ca_.

### 2.3 Optimization of Dosage and Time of ERK and PKC Inhibitors for the Treatment of Cerebral Vessels

These experiments were performed in 8–12 weeks male SD rats. Freshly isolated MCA was cannulated on pre-pulled glass micropipettes (1B120–6, World Precision Instruments, Sarasota, FL), which were mounted in a pressure myograph chamber (Living System Instrumentation, Burlington, VT) as we previously described [41, 42, 47, 48]. The chamber contained PSS solution with 1.6 mM CaCl_2_ and was aerated with air gas (21% O_2_, 5% CO_2_, 74% N_2_) to maintain pH 7.4 at 37 °C. The pressure myograph chamber was connected to an IMT-2 inverted microscope (Olympus, Center Valley, PA) equipped with a digital camera (MU1000, AmScope, Irvine, CA). After gently extending the cannulated MCA to its *in situ* length, any side branches were carefully tied off to prevent any potential leakage. The spontaneous tone of MCA was allowed to equilibrate for 30 minutes with an initial intraluminal pressure of 40 mmHg [49–51]. After preconditioning, the inner diameters (IDs) of MCAs treated with vehicle (0.1% DMSO), FR (30 nM, 300 nM, 1,000 nM), or BIM (3 nM, 30 nM, 300 nM) in response to transmural pressure from 40 to 180 mmHg, in steps of 20 mmHg, were recorded to access the pressure-diameter relationships for optimal dosage. To establish a time curve, the IDs of MCAs treated with vehicle (0.1% DMSO), FR (30 nM, 300 nM, 1,000 nM), or BIM (3 nM, 30 nM, 300 nM) were also recorded at 100 mmHg every 5 minutes for 30 minutes.

### 2.4 Effects of Inhibition of ERK and PKC on the Myogenic Response of MCA and PA in WT and *Dusp5* KO Rats

These experiments were performed in 8–12 weeks male WT and *Dusp5* KO rats. Freshly isolated intact MCA and PA were mounted onto a pressure myograph system as described earlier. The PSS in the bath was maintained at a temperature of 37 °C and a pH of 7.4 by continuously bubbling air. Baseline IDs of the vessels were captured. The MCA and PA were preconditioned at 40 mmHg and 10 mmHg, respectively. The MCA and PA were then treated with vehicle (0.1% DMSO), FR (1,000 nM), or BIM (300 nM) for 10 minutes, and IDs of the MCA and PA in response to pressure from 40 to 180 mmHg and 10 to 60 mmHg, respectively, were recorded. At the end of the experiment, the vessels were washed thoroughly with PSS_0Ca_ under an intraluminal pressure reset at 5 mmHg. The IDs of the MCA and PA were recorded in response to pressure in 20 mmHg increments from 40 to 180 mmHg and 10 to 60 mmHg, respectively, under calcium-free conditions (ID_0Ca_). The values for ID_0Ca_ were obtained by normalizing the IDs of MCA and PA at 40 mmHg and 10 mmHg, respectively, in PSS containing calcium. The myogenic tone is calculated using an equation: myogenic tone (%) = [(ID_0Ca_ – ID)/ ID_0Ca_] X 100 as previously described [28, 29].

### 2.5 Effects of U46619 on the Myogenic Reactivity of MCA and PA in WT and *Dusp5* KO Rats

These experiments were conducted in 8–12 weeks male WT and *Dusp5* KO rats. Freshly isolated intact MCA and PA were mounted onto a pressure myograph system and allowed to equilibrate. Perfusion pressures of 100 mmHg and 30 mmHg were set for MCA and PA, respectively, after preconditioning. The vessels were then treated with U46619 at concentrations ranging from 10^−10^ to 10^−5^ M. The IDs were documented once the vessels had attained a stable state.

### 2.6 Autoregulation of the Surface and Deep Cortical CBF

These experiments were conducted in 8–12 weeks male WT and *Dusp5* KO rats following our published optimized protocol [41–47]. The rats were anesthetized with inactin (50 mg/kg; *i.p.)* and ketamine (30 mg/kg; *i.m.),* and the tracheal, femoral vein and femoral artery were cannulated for connection to a ventilator (SAR-830, CWE Inc.), drug delivery, and blood pressure measurements, respectively. The head of the rat was secured in a stereotaxic device (Stoelting, Wood Dale, IL), and an end-tidal P_CO2_ was maintained at 35 mmHg using a CO_2_ Analyzer (CAPSTAR-100, CWE Inc., Ardmore, PA). A fiber-optic probe (91–00124, Perimed Inc., Las Vegas, NV) connected to a laser Doppler flowmetry device (PF5010, Perimed Inc.) was placed on the cranial window, which was created using a low-speed air drill, and another probe was implanted into the brain for recording the surface and deep cortical regional CBF, respectively. Baseline CBF was recorded at 100 mmHg. The perfusion pressure was then increased to 180 mmHg by infusing phenylephrine (Sigma-Aldrich), followed by a reduction to 40 mmHg by graded hemorrhage in steps of 20 mmHg, and surface and deep cortical CBF was recorded at each pressure level.

### 2.7 Effects of Inhibition of ERK and PKC on Contractile Capability of Cerebral VSMCs Isolated from WT and *Dusp5* KO rats

Primary cerebral VSMCs were isolated from the MCA of 3-week-old male SD, WT and *Dusp5* KO rats, 3–4 rats per strain. The MCA were dissected and digested using a combination of protease papain (22.5 U/mL) and dithiothreitol (2 mg/mL), followed by elastase (2.4 U/mL), collagenase (250 U/mL), and trypsin inhibitor (10,000 U/mL) as we reported [11, 35]. The contractile capabilities of these cells at early passages (P2–4) were evaluated using a collagen gel-based assay kit (CBA-201, Cell Biolabs, San Diego, CA) to assess the effects of ERK and PKC inhibition following our previously optimized protocol [10, 11]. Primary cerebral VSMCs were suspended in the Dulbecco’s Modified Eagle’s Medium (Thermo Scientific, Waltham, MA) supplemented with 20% fetal bovine serum at a density of 2 X 10^6^ cells/mL, which was mixed with a collagen gel working solution on the ice at a ratio of 1:4. In each well of a 24-well plate, a final volume of 500 μL of the cell suspension containing 2 X 10^5^ cells was added for collagen polymerization. After incubating at 37°C for 1 hour, the mixture was added an additional 1 mL of culture medium and further incubated for 2 days at 37°C in a 5% CO_2_ atmosphere to develop contractile stress. To initiate contraction, the stressed matrix was detached from the wall of the well using a sterile needle. At this point, the gel sizes were measured as control values.

To determine the optimal dosage of ERK and PKC inhibitors for treating cerebral VSMCs, we used cells isolated from SD rats. After the initial contraction, 1 mL of DMEM containing vehicle (0.1% DMSO), FR (100 nM, 1,000 nM, 10,000 nM), or BIM (30 nM, 300 nM, 3,000 nM), respectively, was added on top of the collagen gel. The changes in collagen gel size were imaged every 30 minutes for 2 hours and quantified using PowerPoint 2016 (Microsoft Corporation, Redmond, WA). Experiments were performed in triplicate and repeated three times.

To determine the effects of ERK and PKC inhibition on the contractile capabilities of primary cerebral VSMCs isolated from WT and *Dusp5* KO rats, we included vehicle (0.1% DMSO), FR (1,000 nM), or BIM (300 nM) in 1 mL of DMEM, which was then applied on top of the cell-gel mixture after the initial contraction. The changes in collagen gel size were imaged and quantified at 120 minutes. The experiments were performed in triplicate and repeated three times.

### 2.8 Eight-arm Water Maze

To assess hippocampal-based spatial learning and memory, we conducted an eight-arm water maze experiment using a protocol previously published by our research group [41, 52, 53]. Six months old male WT and *Dusp5* KO rats were first pre-trained to recognize and memorize the escape platform. Then, we evaluated their performance in eight trials, conducted at 2 and 24 hours after the training phase. We recorded the time taken by each animal to reach the platform, which we presented as the escape time.

### 2.9 Statistical Analyses

Data are presented as mean values ± standard error of the mean (SEM). For comparing differences in continuously measured groups, a two-way ANOVA for repeated measures followed by a Holm-Sidak *post hoc* test was used. To evaluate the significance of differences between two groups or treatments, a paired or unpaired *t*-test, or a one-way ANOVA followed by a *post hoc* Tukey test was used. GraphPad Prism 9 (GraphPad Software, Inc., La Jolla, CA) was used for statistical analysis. Results were considered statistically significant if *P* < 0.05.

## 3. Results

### 3.1 Optimization of Dosage and Time of ERK and PKC Inhibitors for the Treatment of Cerebral Vessels

**Figure 1:**
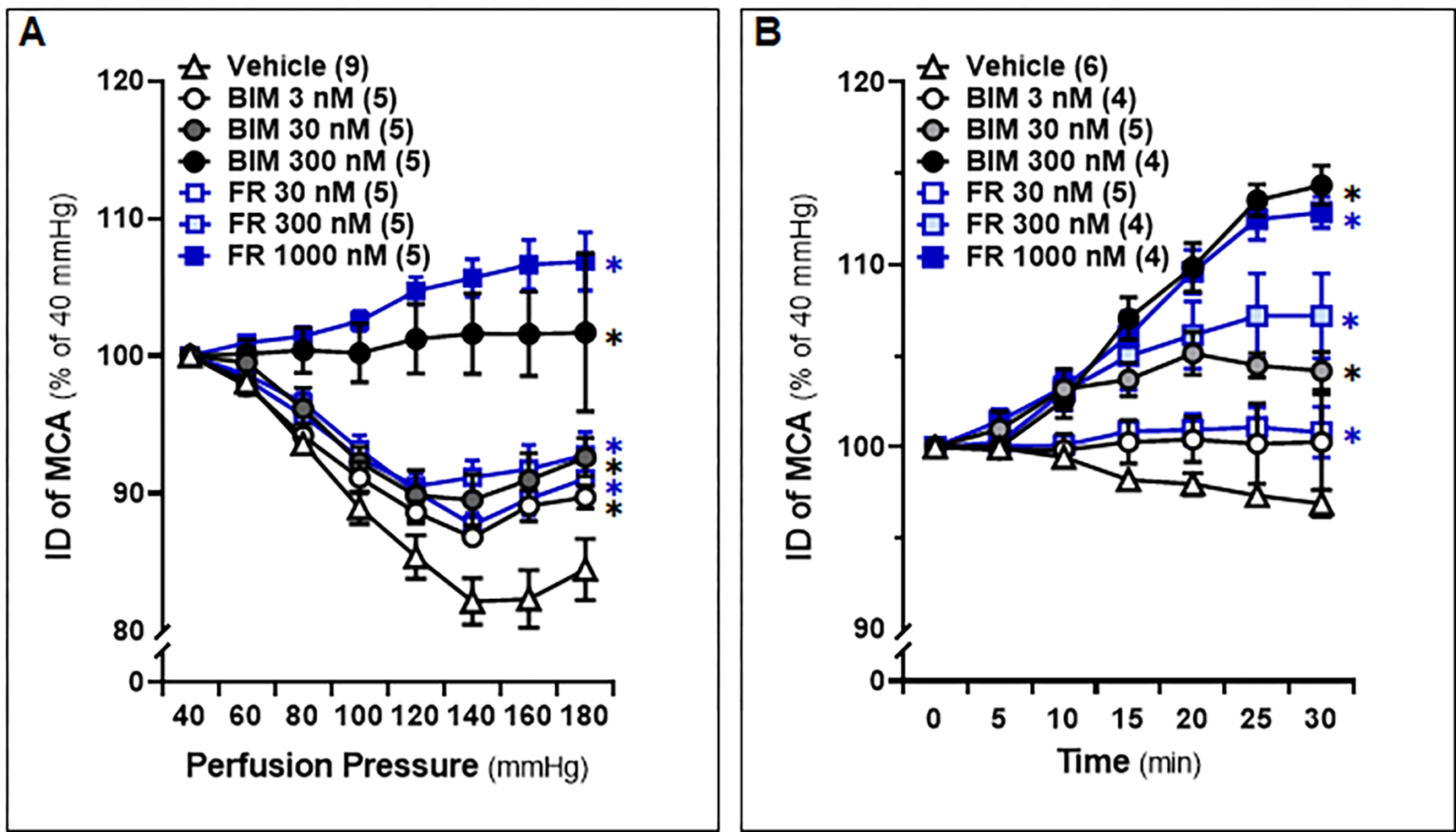
Optimization of Dosage and Time of Extracellular Signal-Regulated Kinase (ERK) and Protein Kinase C (PKC) Inhibitors for the Treatment of Cerebral Vessels Middle cerebral arteries (MCAs) were freshly isolated from 8–12 weeks old male Sprague-Dawley (SD) rats and treated with vehicle (0.1% DMSO), FR180204 (FR; 30 nM, 300 nM, 1,000 nM), or bisindolylmaleimide III (BIM; 3 nM, 30 nM, 300 nM). **A.** Comparison of the inner diameters (IDs) of the MCA in response to intramural pressures from 40 to 180 mmHg. **B.** Time course of the changes in vascular IDs. The IDs of the MCA at intramural pressure of 100 mmHg were measured every 5 minutes for 30 minutes. The numbers in parentheses indicate the number of rats studied per group. The mean values ± SEM are presented, and * denotes *P* < 0.05 from the corresponding values in vehicle-treated vessels.

The MCA freshly isolated from 8–12 weeks male SD rats and treated with 0.1 % DMSO constricted by 17.9 ± 1.7% upon increasing perfusion pressure from 40 to 140 mmHg, and slightly dilated by 16.2 ± 2.4% at 180 mmHg, which was similar to non-treated vessels [48]. In contrast, IDs of both FR and BIM-treated vessels exhibited a dose-dependent increase in response to pressure changes from 40 to 180 mmHg (Fig. 1A). MCAs treated with FR (1,000 nM) or BIM (300 nM) exhibited significant dilation, starting at 10 minutes of treatment under a physiological pressure of 100 mmHg, and this dilation continued to extend until 30 minutes of treatment. (Fig. 1B). Based on these results, we incubated cerebral vessels with FR (1,000 nM) or BIM (300 nM) for 10 minutes before evaluating the effects of these inhibitors on the myogenic responses in the MCA and PA of WT and *Dusp5* KO rats in subsequent experiments.

### 3.2 Effects of Inhibition of ERK and PKC on the Myogenic Response of MCA in WT and *Dusp5* KO Rats

**Figure 2:**
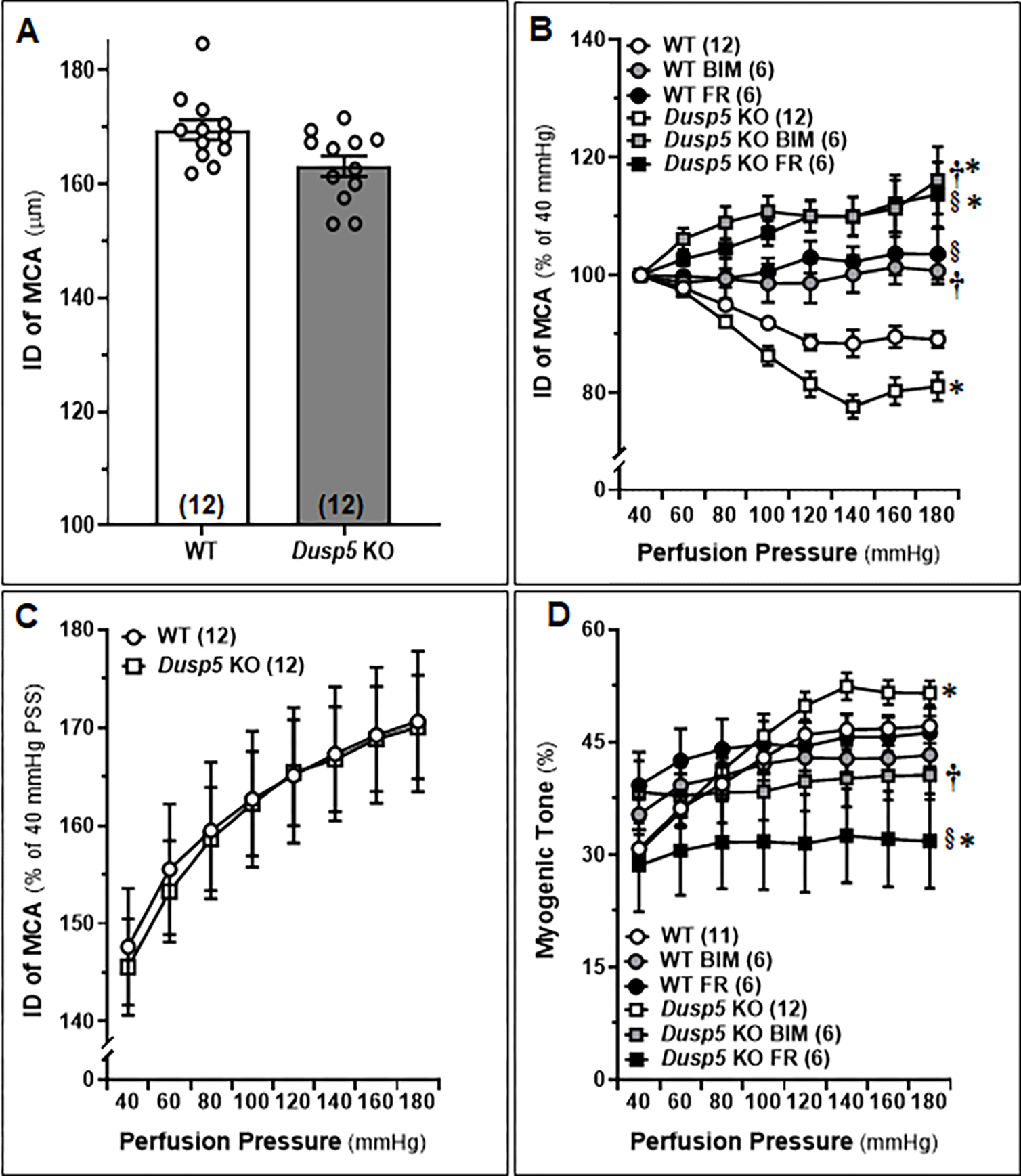
Effects of Inhibition of Extracellular Signal-Regulated Kinase (ERK) and Protein Kinase C (PKC) on the Myogenic Response of Middle Cerebral Arteries (MCAs) in Wildtype (WT) and *Dusp5* KO Rats MCAs were freshly isolated from 8–12 weeks male WT and *Dusp5* KO rats and treated with vehicle (0.1% DMSO), FR180204 (FR; 1,000 nM), or bisindolylmaleimide III (BIM; 300 nM). **A.** Comparison of baseline inner diameters (IDs) of the MCA from WT and *Dusp5* KO rats. **B.** Comparison of the IDs of the MCAs of WT and *Dusp5* KO rats with various treatments at intramural pressure of 100 mmHg, measured every 5 minutes for 30 minutes. **C.** ID measured under calcium-free conditions (ID_0Ca_) of the MCA of WT and *Dusp5* KO rats, measured in calcium-free physiological salt solution (PSS), was compared by normalizing the IDs of MCA at 40 mmHg in PSS containing calcium. **D.** Comparison of the myogenic tone of the MCA of WT and *Dusp5* KO rats with various treatments. The numbers in parentheses indicate the number of rats studied per group. The mean values ± SEM are presented, and * denotes *P* < 0.05 from the corresponding values obtained from WT vessels, **†** denotes *P* < 0.05 from the corresponding values obtained from BIM-treated vessels within the strain, § denotes *P* < 0.05 from the corresponding values obtained from FR-treated vessels within the strain.

The baseline IDs of the MCA of 8–12 weeks old male WT and *Dusp5* KO rats were no different (Fig. 2A). Both FR (1,000 nM) and BIM (300 nM) treatments resulted in significant dilation of MCA in *Dusp5* KO rats, starting at 80 mmHg with dilation levels of 4.54 ± 1.6% and 8.94 ± 1.7%, respectively. On the other hand, FR (1,000 nM) and BIM (300 nM) treatments led to slight dilation in MCA of WT rats, starting from 100 mmHg and 140 mmHg, respectively. The inhibition of ERK and PKC had a more pronounced effect on vessels from *Dusp5* KO rats compared to those from WT rats (Fig. 2B). Furthermore, we found that the values for ID_0Ca_ were similar in response to perfusion pressures from 40 to 180 mmHg between *Dusp5* KO and WT rats (Fig. 2C). As presented in Fig. 2D, the myogenic tone was similar in *Dusp5* KO and WT rats in the pressure range of 40 to 80 mmHg. However, the tone became higher in *Dusp5* KO rats in the pressure range of 100 to 180 mmHg. Both FR (1,000 nM) and BIM (300 nM) treatments resulted in significantly reduced myogenic tone in *Dusp5* KO rats but not in WT rats, compared to vehicle-treated vessels.

### 3.3 Effects of Inhibition of ERK and PKC on the Myogenic Response of PAs in WT and *Dusp5* KO Rats

**Figure 3:**
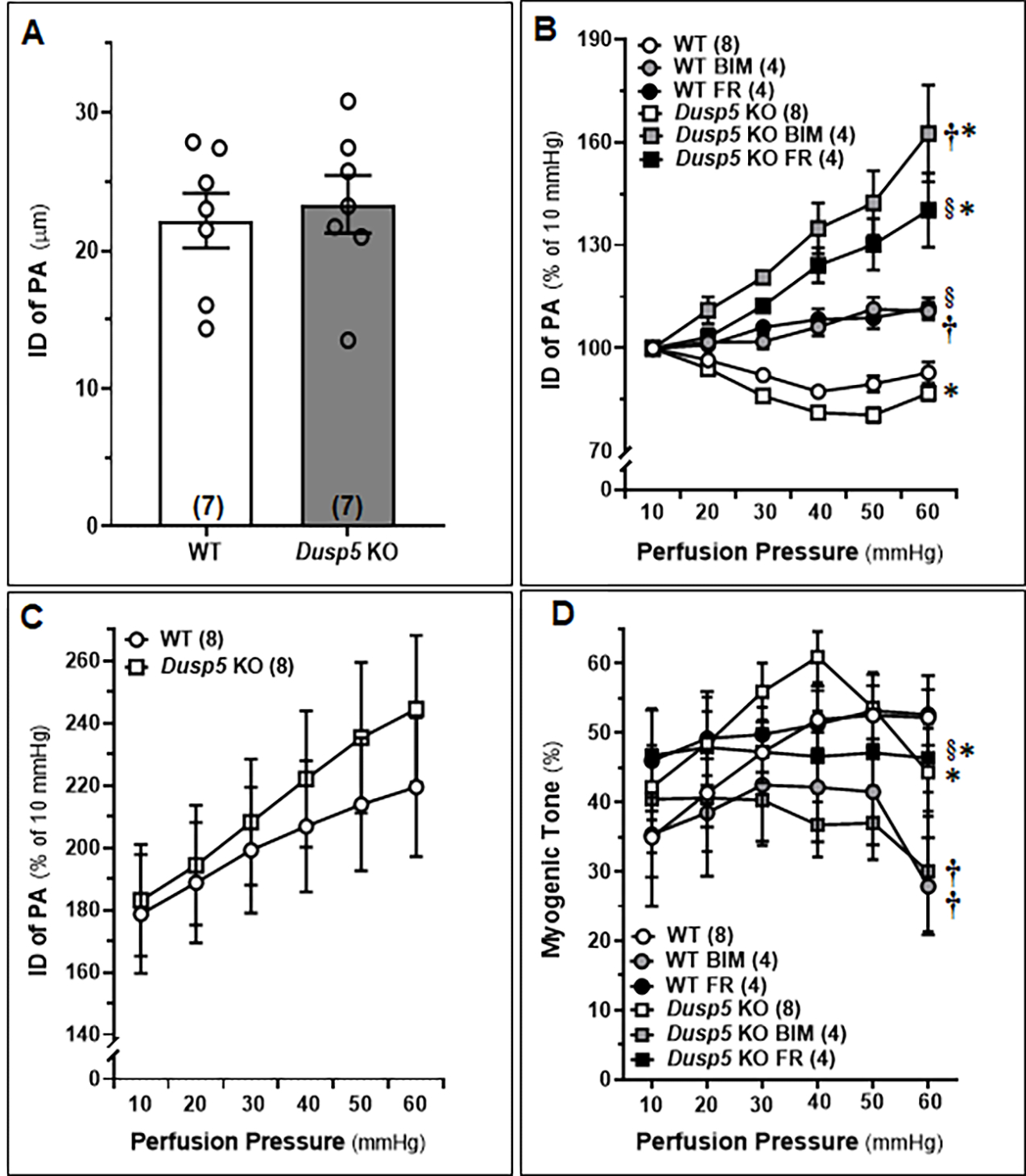
Effects of Inhibitors of Extracellular Signal-Regulated Kinase (ERK) and Protein Kinase C (PKC) on the Myogenic Response and Myogenic Tone in the Penetrating Arterioles (PAs) of *Dusp5* KO rats PAs were freshly isolated from 8–12 weeks male wildtype (WT) and *Dusp5* KO rats and treated with vehicle (0.1% DMSO), FR180204 (FR; 1,000 nM), or bisindolylmaleimide III (BIM;300 nM). **A.** Comparison of baseline inner diameters (IDs) of the PAs from WT and *Dusp5* KO rats. **B.** Comparison of the IDs of the PAs of WT and *Dusp5* KO rats with various treatments at intramural pressure of 30 mmHg, measured every 5 minutes for 30 minutes. **C.** ID measured under calcium-free conditions (ID_0Ca_) of the PAs of WT and *Dusp5* KO rats, measured in calcium-free physiological salt solution (PSS), was compared by normalizing the IDs of PAs at 10 mmHg in PSS containing calcium. **D.** Comparison of the myogenic tone of the PAs of WT and *Dusp5* KO rats with various treatments. The numbers in parentheses indicate the number of rats studied per group. The mean values ± SEM are presented, and * denotes *P* < 0.05 from the corresponding values obtained from WT vessels, **†** denotes *P* < 0.05 from the corresponding values obtained from BIM-treated vessels within the strain, § denotes *P* < 0.05 from the corresponding values obtained from FR-treated vessels within the strain.

The baseline IDs of the MCA of male WT and *Dusp5* KO rats aged 8–12 weeks showed no significant difference (Fig. 3A). Upon treatment with FR (1,000 nM) and BIM (300 nM), significant dilation of PAs was observed in *Dusp5* KO rats, starting at 20 mmHg with dilation levels of 3.20 ± 2.5% and 11.03 ± 3.9%, respectively. Similarly, significant dilation of PAs in WT rats was observed upon treatment with FR (1,000 nM) and BIM (300 nM), starting from 20 mmHg with dilation levels of 0.99 ± 1.0% and 1.73 ± 1.8%, respectively. (Fig. 3B). These results suggest that the inhibition of ERK and PKC had a more pronounced effect on vessels from *Dusp5* KO rats compared to those from WT rats. The values for ID_0Ca_ were found to be similar in response to perfusion pressures ranging from 10 to 60 mmHg between *Dusp5* KO and WT rats (Fig. 3C). Consistent with our previous report, [28] myogenic tone was higher in *Dusp5* KO compared to WT rats in the pressure range of 10 to 40 mmHg but became similar at 50 mmHg and lower in *Dusp5* KO than in WT rats at 60 mmHg (Fig. 3D). Treatment with both FR (1,000 nM) and BIM (300 nM) resulted in a significant reduction of myogenic tone in vessels from *Dusp5* KO rats and in WT rats, compared to those treated with vehicle.

### 3.4 Autoregulation of the Surface and Deep Cortical CBF

**Figure 4:**
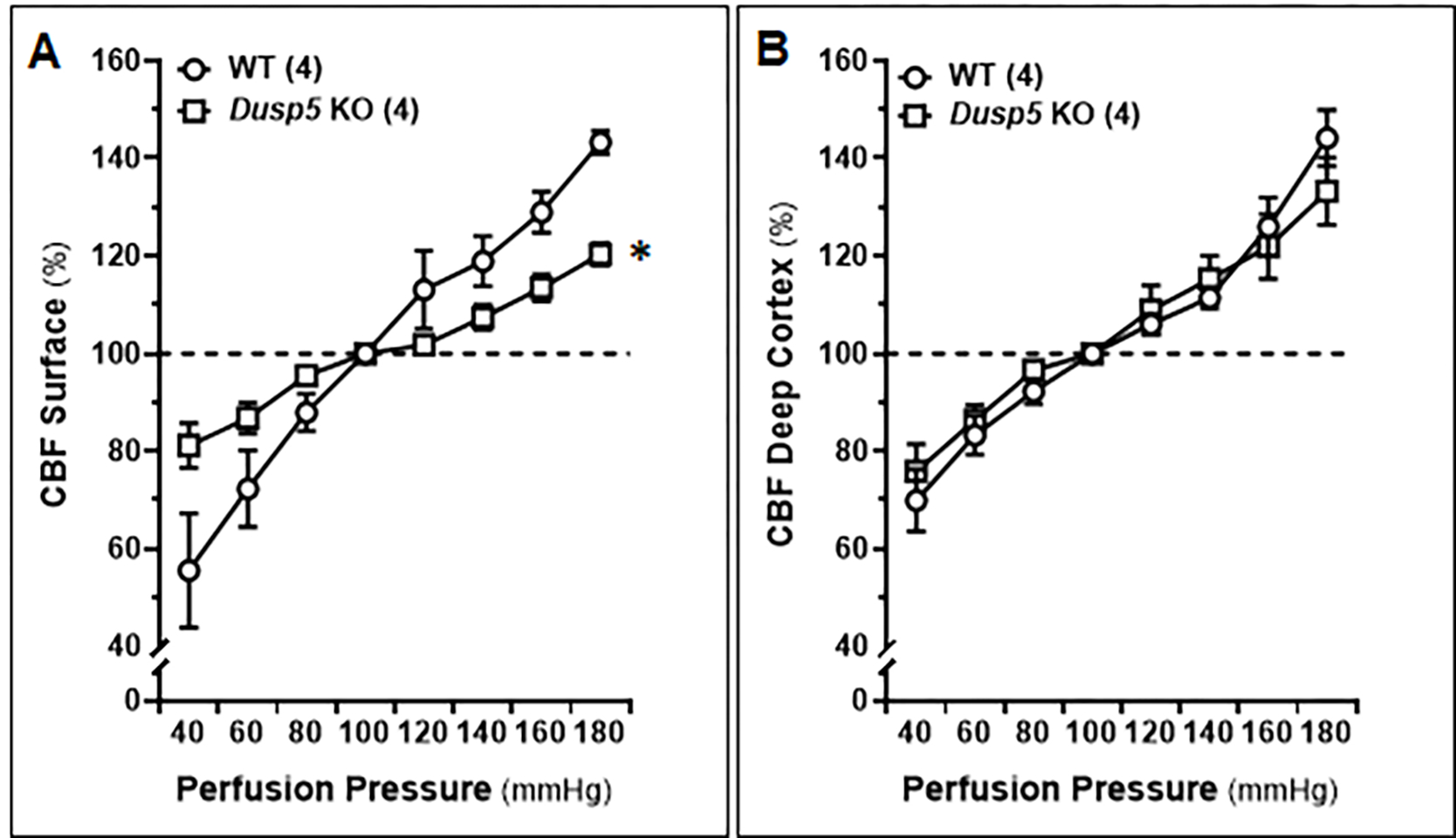
Autoregulation of the Surface and Deep Cortical Cerebral Blood Flow (CBF) Comparison of the autoregulation of surface and deep cortical CBF in 8–12 weeks old male wildtype (WT) and *Dusp5* KO rats. **A**. Comparison of surface cortical CBF autoregulation as of % to 100 mmHg. **B.** Comparison of deep cortical CBF autoregulation as of % to 100 mmHg. The numbers in parentheses indicate the number of rats studied per group. The mean values ± SEM are presented, and * indicates a significant difference (*P* < 0.05) from the corresponding values in age-matched WT rats.

We compared the autoregulation of surface and deep cortical CBF in male WT and *Dusp5* KO rats aged 8–12 weeks. Our results, as shown in Fig. 4A, demonstrate that *Dusp5* KO rats exhibited stronger surface cortical CBF autoregulation compared to WT rats. Specifically, when perfusion pressure increased from 100 to 140 mmHg, the CBF increased by 5.75 ± 1.06% in *Dusp5* KO rats, while it increased by 21.78 ± 2.11% in WT rats. Conversely, when perfusion pressure decreased from 100 to 60 mmHg, the CBF decreased by 12.97 ± 2.46% in *Dusp5* KO rats, whereas it decreased by 32.15 ± 5.81% in WT rats. Interestingly, our results also showed that the deep cortical CBF autoregulation was similar in both WT and *Dusp5* KO rats. Specifically, when perfusion pressure increased from 100 to 140 mmHg, the CBF increased by 15.53 ± 5.05% in *Dusp5* KO rats and 12.61 ± 2.44% in WT rats. Similarly, when perfusion pressure decreased from 100 to 60 mmHg, the CBF decreased by 15.98 ± 3.9% in *Dusp5* KO rats and 16.71 ± 3.99% in WT rats (Fig. 4B).

### 3.5 Effects of Inhibition of ERK and PKC on Contractile Capability of Cerebral VSMCs Isolated from WT and *Dusp5* KO Rats.

**Figure 5:**
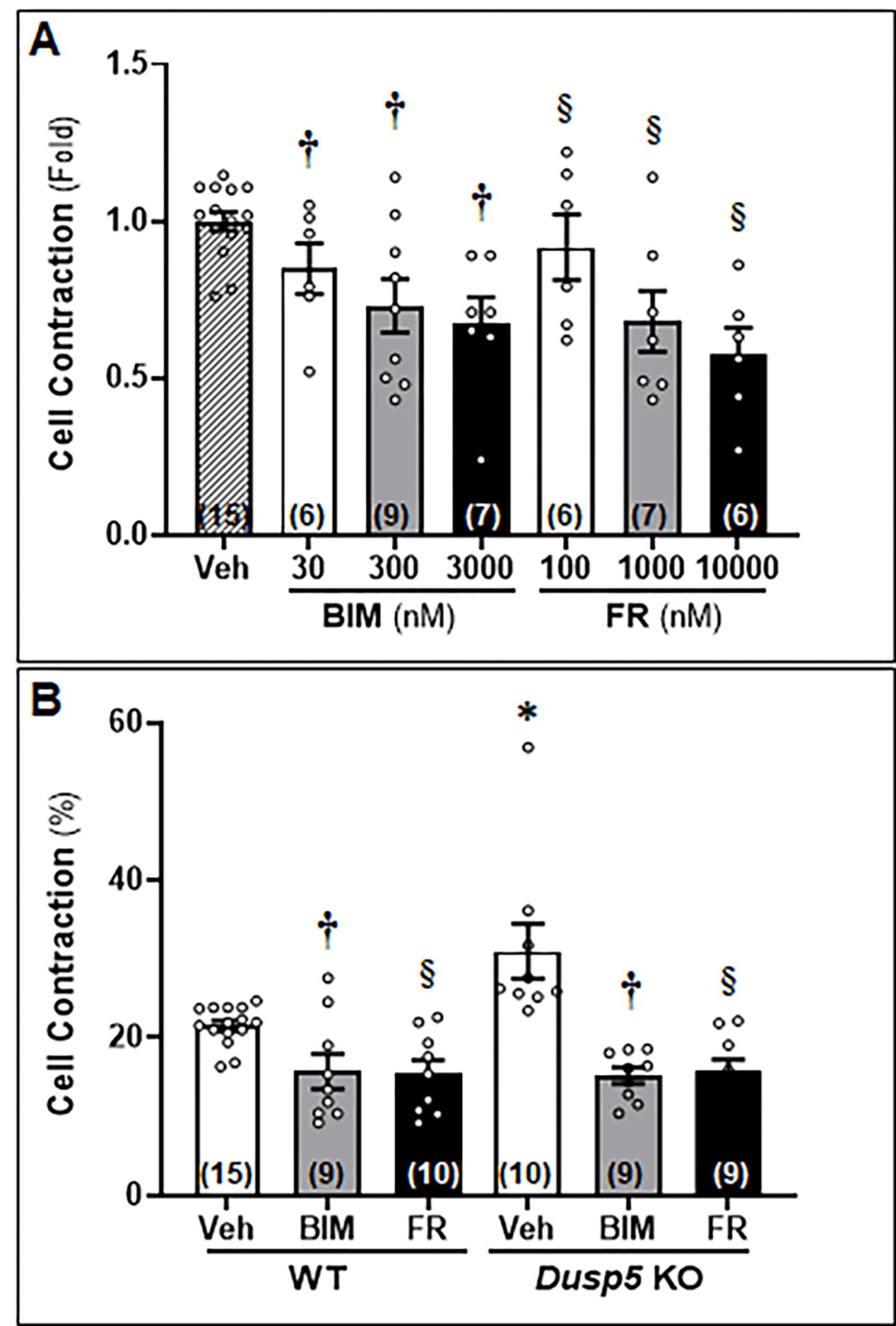
Effects of Inhibition of Extracellular Signal-Regulated Kinase (ERK) and Protein Kinase C (PKC) on Contractile Capability of Primary Cerebral Vascular Smooth Muscle Cells (VSMCs) Isolated from Wildtype (WT) and *Dusp5* KO rats To assess the impact of FR180204 (FR) and bisindolylmaleimide III (BIM) on the contractile capacity of VSMCs in *Dusp5* KO rats as compared to WT rats, we first optimized the dosage and duration of the treatment using cerebral VSMCs isolated from Sprague-Dawley (SD) rats. As depicted in Fig. 5A, vessels treated with both FR and BIM exhibited a reduction in contraction that was dose-dependent, as compared to cells treated with the vehicle. Fig. 5B demonstrates that *Dusp5* KO cells treated with the vehicle displayed a contraction of 30.97 ± 3.49%, which was significantly higher than that of the vehicle-treated WT cells (21.53 ± 0.64%). However, both BIM and FR-treated *Dusp5* KO cells showed a significant decrease in contractile capability compared to the vehicle-treated cells, with contractions of 15.21 ± 1.02% and 15.85 ± 1.40%, respectively. Similarly, both BIM and FR-treated WT cells exhibited a significant increase in contractile capability compared to the vehicle-treated cells, with contractions of 15.76 ± 2.20% and 15.49 ± 1.71%, respectively. There were no differences observed between the strains in the same treatment groups. * denotes *P* < 0.05 from the corresponding values obtained from untreated cells, **†** denotes *P* < 0.05 in BIM-treated cells from the corresponding values obtained from untreated cells, § denotes *P* < 0.05 in FR-treated cells from the corresponding values obtained from non-FR-treated cells.

To assess the impact of FR and BIM on the contractile capacity of VSMCs in *Dusp5* KO rats as compared to WT rats, we first aimed to optimize the dosage and duration of the treatment using cerebral VSMCs isolated from SD rats. As depicted in Fig. 5A, vessels treated with both FR and BIM exhibited a reduction in contraction that was dose-dependent, as compared to cells treated with the vehicle. Fig. 5B demonstrates that *Dusp5* KO cells treated with the vehicle displayed a contraction of 30.97 ± 3.49%, which was significantly higher than that of the vehicle-treated WT cells (21.53 ± 0.64%). However, both BIM and FR-treated *Dusp5* KO cells showed a significant decrease in contractile capability compared to the vehicle-treated cells, with contractions of 15.21 ± 1.02% and 15.85 ± 1.40%, respectively. Similarly, both BIM and FR-treated WT cells exhibited a significant decrease in contractile capability compared to the vehicle-treated cells, with contractions of 15.76 ± 2.20% and 15.49 ± 1.71%, respectively. There were no differences observed between the strains in the same treatment groups.

### 3.6 Effects of U46619 on the Myogenic Reactivity of MCA and PAs in WT and *Dusp5* KO Rats

**Figure 6:**
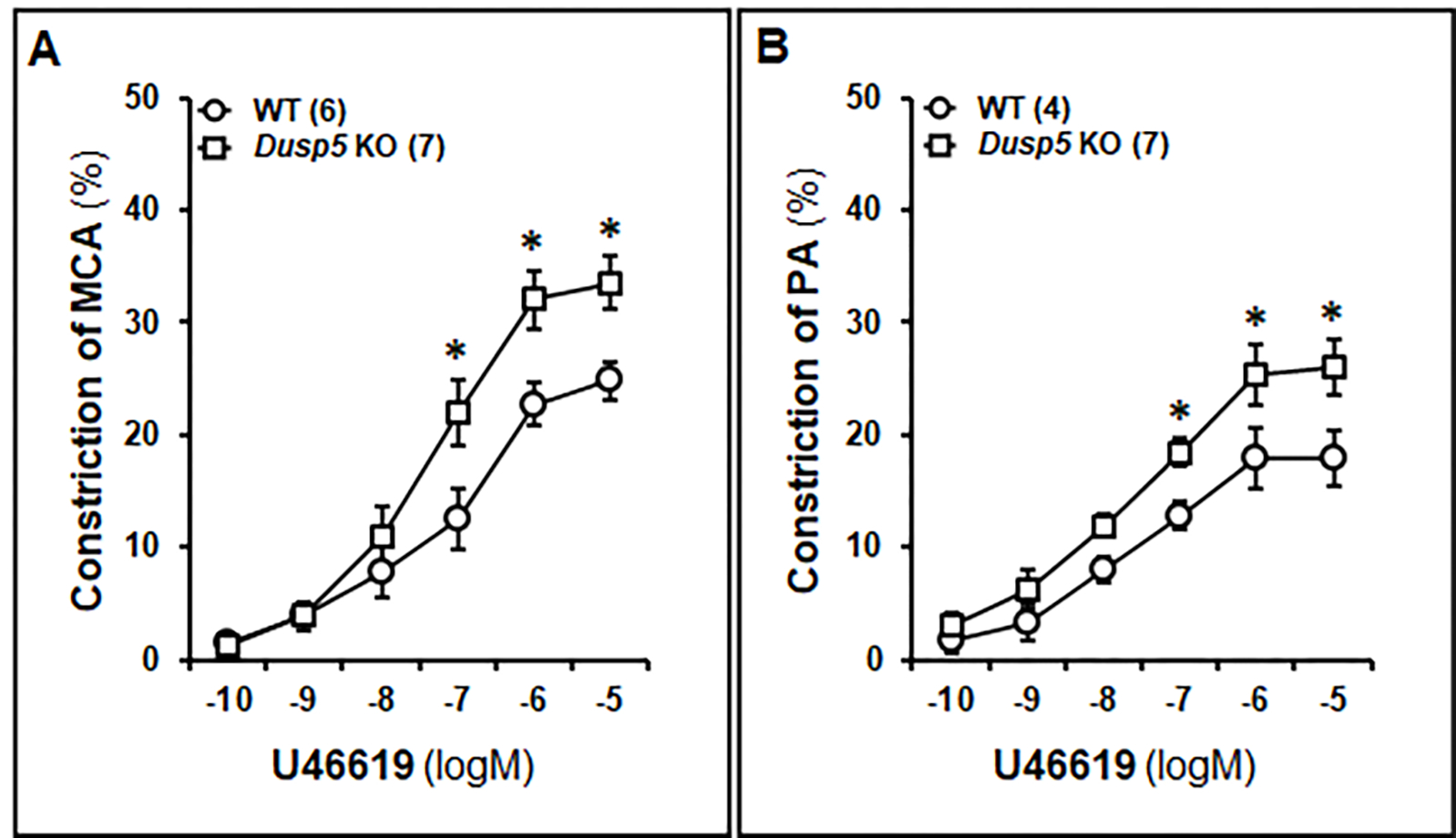
Effects of U46619 on the Myogenic Reactivity of Middle Cerebral Arteries (MCAs) and Penetrating Arterioles (PAs) in Wildtype (WT) and *Dusp5* KO Rats MCAs and PAs were freshly isolated from 8–12 weeks male WT and *Dusp5* KO rats and treated with U46619 at concentrations ranging 10^−10^ to 10^−5^ M. **A.** Comparison of the dose-response of constriction (%) of the MCA from WT and *Dusp5* KO rats. **B.** Comparison of the dose-response of constriction (%) of the PAs from WT and *Dusp5* KO rats. The numbers in parentheses indicate the number of rats studied per group. The mean values ± SEM are presented, and * denotes *P* < 0.05 from the corresponding values obtained from WT vessels.

As demonstrated in Fig. 6A, U46619-treated MCAs in both WT and *Dusp5* KO rats exhibited a dose-dependent decrease in diameter. The concentration-response curves were shifted to the left in both the MCA and PA in *Dusp5* KO rats. Notably, at higher concentrations (10^−7^ to 10^−5^ M), *Dusp5* KO vessels displayed a significantly greater reduction in diameter than WT vessels, with a maximal decrease of 33.58 ± 2.45% and 24.82 ± 1.74%, respectively. Likewise, U46619-induced constriction of PAs in both WT and *Dusp5* KO rats displayed a dose-dependent reduction in IDs. At higher concentrations (10^−7^ to 10^−5^ M), *Dusp5* KO vessels potentiated the vasoconstriction response than WT vessels, with a maximal ID reduction of 25.94 ± 2.48% and 17.91 ± 0.98%, respectively. (Fig. 6B).

### 3.7 Eight-arm Water Maze

**Figure 7:**
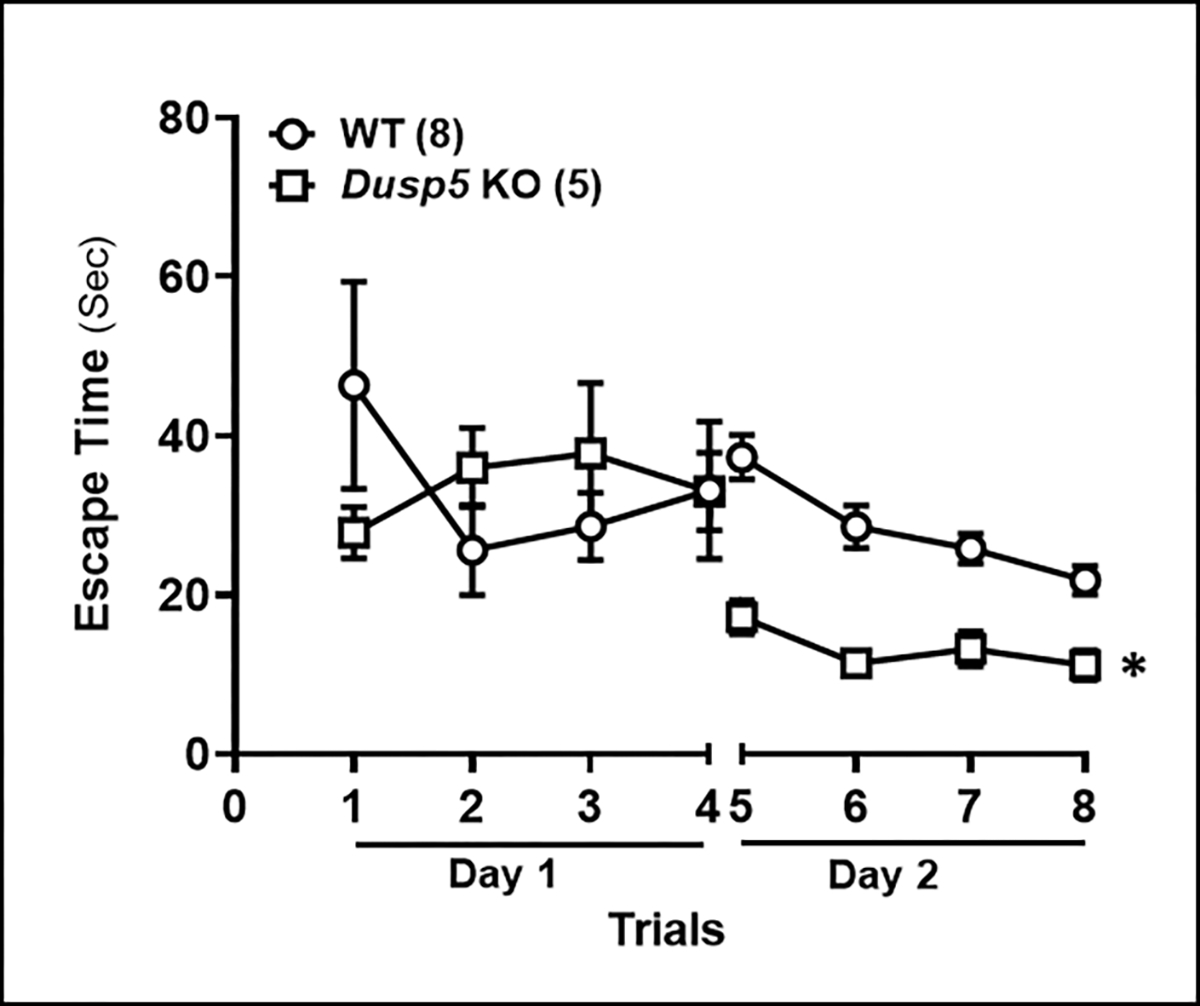
Eight-arm Water Maze Comparison of the time to reach the platform per trial between 6 months old WT and *Dusp5* KO rats. The numbers in parentheses indicate the number of rats studied per group. The mean values ± SEM are presented, and * denotes *P* < 0.05 from the corresponding values obtained from WT vessels.

We found no significant difference in escape time between the two strains of rats on the first day. However, 24 hours after training, *Dusp5* KO rats demonstrated a significantly shorter escape time compared to age-matched WT rats (Fig. 7).

## 4. Discussion

The present study builds on our previous findings, which revealed enhanced myogenic response and autoregulation in the cerebral and renal circulations of *Dusp5* KO rats associated with increased levels of phosphorylated PKCα and ERK1/2 [26–28]. The current findings expand upon these results by demonstrating that inhibition of ERK and PKC leads to a dose-dependent dilation of MCA and PA, and a reduction in the myogenic responses of these vessels. We observed that both ERK and PKC inhibitors, as well as the thromboxane A2 agonist U46619, had a more pronounced effect on MCA and PA vessels from *Dusp5* KO rats compared to those from WT rats. Although baseline IDs were comparable between the two strains under physiological pressure, the MCA of *Dusp5* KO rats exhibited greater myogenic tone within the pressure range of 100 to 180 mmHg. In addition, the myogenic tone of PA in *Dusp5* KO rats showed a unique pattern, with higher tone at lower perfusion pressures, peaking at 40 mmHg, and decreasing at 60 mmHg, consistent with our previous report [28]. Both ERK and PKC inhibitors resulted in a significant reduction of myogenic tone in both MCA and PA from *Dusp5* KO rats. *We also* revealed that *Dusp5* KO rats exhibited better autoregulation of surface but not deep cortical CBF when compared to WT rats. These findings suggest that altered vascular reactivity in *Dusp5* KO rats may involve distinct mechanisms for different vascular beds, underscoring the need for further investigation to understand the underlying mechanisms. Moreover, we explored the effect of inhibiting ERK and PKC on the contractile ability of cerebral VSMCs in both WT and *Dusp5* KO rats. The result that Gq-phospholipase C-PKC-calcium dependent vasoconstrictor response of the MCA and PA to U46619 was potentiated in *Dusp5* KO rats is consistent with our previous report that vascular pPKC levels are elevated in this strain [26–28]. This result is also consistent with previous reports that the response of human cerebral arteries to endothelin-1, 5-hydroxytryptamine, TXA_2_ and angiotensin II are all modulated by the MAPK signaling that is regulated by DUSP 5 [54]. Our findings demonstrate that treatment with BIM and FR significantly enhanced the contractile capacity of VSMCs from both strains. Finally, we compared cognitive function between WT and *Dusp5* KO rats and observed a significant improvement in learning and memory in *Dusp5* KO rats 24 hours after initial training. These results suggest that DUSP5 may play a role in cognitive function, and its inhibition may have therapeutic potential in cognitive disorders, such as AD/ADRD.

Reduction in CBF, as one of the early symptoms of AD/ADRD has been observed in numerous human and animal models of the disease [1, 2, 41]. The strong correlation between the severity of brain hypoperfusion and the extent of cognitive deficits [3, 4] underscores the importance of studying this phenomenon. Understanding the underlying mechanisms of impaired vascular reactivity and CBF reduction is essential for the development of effective interventions and therapies to prevent or delay cognitive decline in AD/ADRD. Impaired myogenic response and CBF autoregulation are among the vascular mechanisms that contribute to brain hypoperfusion [2, 5]. Capillary pericyte constriction induced by Aβ has been shown to reduce capillary IDs in AD, [55] but it is uncertain whether this small diameter reduction is a compelling explanation for significantly reduced perfusion. Furthermore, capillary pericyte constriction can also be induced by hyperoxia, [56] which may be a consequence of hypoperfusion. On the other hand, brain hypoperfusion also exacerbates AD pathology and cognitive impairment [57]. It is difficult to determine if Aβ or brain hypoperfusion is a cause or consequence of AD/ADRD [58]. However, brain hypoperfusion and Aβ accumulation may mutually reinforce each other, promoting the development of cognitive impairments in AD.

The DUSP family is implicated in the development and progression of AD/ADRD [30]. Evidence suggests that DUSP5 may play an essential role in regulating the plasticity of the hippocampal dentate gyrus, [31] a brain region critical for learning and memory. Additionally, *DUSP5* has been demonstrated to be associated with late-onset AD, [32] particularly in APOE ε4 non-carrier patients. ^33^ Given the importance of hippocampal dysfunction in AD/ADRD and strong evidence in human studies, understanding the specific role of DUSP5 in AD pathogenesis - including its potential independence from Aβ aggregation, neurofibrillary tangle formation, and synaptic plasticity impairment - may provide valuable insights into the development and treatment of these disorders.

Interestingly, the enhanced CBF autoregulation was observed only on the surface but not in deep cortical regions. This lack of effect in the deep cortical regions may be due to the eutrophic vascular hypotrophy and higher myogenic tone at lower pressures found in the PAs of *Dusp5* KO rats, which dropped at high pressures. Our findings that KO of *Dusp5* enhances myogenic response and autoregulation of CBF implies that DUSP5 could potentially contribute to AD pathogenesis by regulation of brain perfusion. We confirmed this hypothesis and found that the learning and memory in *Dusp5* KO rats were improved 24 hours after initial training.

Indeed, the present study has limitations. For instance, the WT rat is not a cognitive impairment model. The FHH.1^BN^ congenic rat was created by transferring a small segment of chromosome 1 from the Brown Norway rat into the Fawn Hooded hypertensive (FHH) genetic background, which contained 15 genes including *Dusp5*, [26, 37] although we have reported that the FHH rat is a potential ADRD model [59–62]. Future study is necessary to confirm whether DUSP5 expression is elevated in models of AD/ADRD, and whether the downregulation of DUSP5 expression or the use of DUSP5 inhibitors, such as compounds containing a naphthalene trisulfonate core, [63] or halogenated xanthene inhibitors of DUSP5, can enhance CBF and cognitive function in individuals with AD/ADRD [64]. Capillary stalling is another vascular mechanism that contributes to cerebral hypoperfusion in AD by causing neutrophil arrest and increasing the expression of inflammatory adhesion molecules in capillaries [6]. It is plausible that the KO of *Dusp5* may also mitigate macrophage infiltration, as we observed in the kidney following hypertension induction in this model [27]. Furthermore, it is worth exploring whether the KO of *Dusp5* reduces BBB permeability and neurodegeneration in aging, hypertension, or diabetes as we observed in other models [65, 66].

To conclude, our findings provide evidence that targeting DUSP5 could be a promising approach for ameliorating cerebral hypoperfusion and cognitive impairment in AD/ADRD. Future studies should examine the therapeutic effects of DUSP5 inhibitors in preclinical and clinical models of AD/ADRD.
